# The binding assessment with human serum albumin of novel six-coordinate Pt(IV) complexes, containing bidentate nitrogen donor/methyl ligands

**Published:** 2015-12

**Authors:** Reza Yousefi, Asghar Taheri-Kafrani, Sayed Masoud Nabavizadeh, Zahra Pouryasin, Mohammad Bagher Shahsavani, Kazem Khoshaman, Mehdi Rashidi

**Affiliations:** 1 Protein Chemistry Laboratory (PCL), Department of Biology, College of Sciences, Shiraz University, Shiraz, Iran; 2 Department of Biotechnology, Faculty of Advanced Sciences and Technologies, University of Isfahan, Isfahan, 81746-73441, Iran; 3 Department of Chemistry, College of Sciences, Shiraz University, Shiraz, Iran

**Keywords:** Human serum albumin, Platinum (IV) complexes, Spectroscopic studies, Molecular docking simulation

## Abstract

The interactions between platinum complexes and human serum albumin (HSA) play crucial roles in the distribution, metabolism, and activity of platinum-based anticancer drugs. Octahedral platinum (IV) complexes represent a significant class of anticancer agents that display molecular pharmacological properties different from cisplatin. In this study, the interaction between two Pt(IV) complexes with the general formula [Pt(X)2Me2 (tbu2bpy)], where tbu2bpy = 4,4′-ditert-butyl-2,2′-bipyridine, with two leaving groups of X = Cl (**Com1**) or Br (**Com2**), and HSA were investigated, using Ultraviolet-Visible (UV-Vis) spectroscopy, fluorescence spectroscopy, circular dichroism (CD) and molecular docking simulation. The spectroscopic and thermodynamic data revealed that the HSA/Pt(IV) complexes interactions were spontaneous process and **Com2 **demonstrated stronger interaction and binding constant in comparison with **Com1**. Also, the results suggest approximately similar structural alteration of HSA in the presence of these Pt complexes. Molecular docking revealed that both Pt(IV) complexes bind with HSA in subdomain IB, literally the same as each other. This study suggests that variation in the leaving group, displaying differing departure rate, has no significant contribution in denaturing prosperities of the Pt(IV) complexes against HSA.

## INTRODUCTION

Recently, the systemic toxicity and cellular resistance are two foremost insufficiencies of the platinum-based anticancer drugs [1]. Although, genomic DNA isthe main cellular target of platinum anticancer drugs, their non-specific interactions with other biomolecules e.g. human plasma proteins have been considered as the most likely cause of their poor therapeutic index and the main reason for development of various side effects and resistance [2]. One of the important approaches to overcome these complications in the clinical practice is to search for non-classical structural innovations which violate the empirical structure-activity rules (SAR) of the conventional platinum drugs [3].The platinum (II) anticancer drugs with the square-planar geometry and high reactivity are believed to have also poor biological stability and lower solubility. On administration to the cancer patients, the platinum (II) anticancer drugs may demonstrate very fast and non-selective reactions with a wide variety of non-specific biological target molecules, before reaching the genomic DNA. Therefore, a keen interest has been given newly to the octahedral Pt(IV) complexes which due to their greater chemical stability, display significant potential advantages, allowing a larger amount of the drugs to arrive at the target tissue for interaction with the genomic DNA, without reacting with other biomolecules [4]. Because of the chemical inertness, Pt(IV) complexes generally serve as prodrug and can be activated by the reducing environment of the tumor tissue which convert the octahedral Pt(IV) to square planar Pt(II) via a two electron reduction and loss of the axial leaving groups [5].

**Figure 1:**
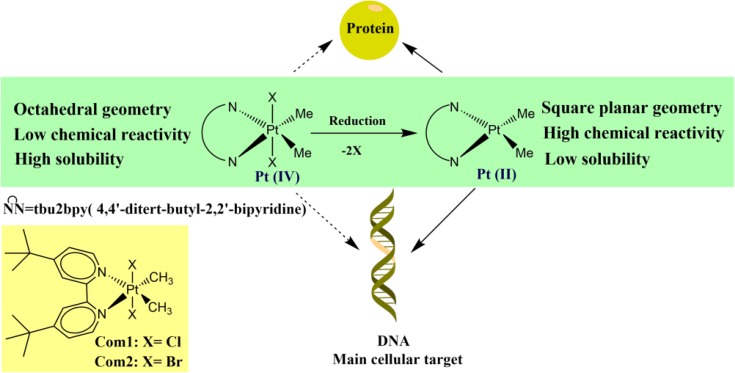
The activation of Pt (IV) complex under reducing environment. Pt (IV) complexes which generally serve as prodrugs, loss their leaving groups in the reducing environment and subsequently convert into Pt (II) complexes which demonstrate high reactivity toward DNA as the main molecular target and other biomolecules including proteins. Also, in this figure the molecular structures of the synthesized complexes (Com1 and Com2) are indicated

Recently, some Pt(IV) complexes with relatively high antitumor activity and low renal toxicity (e.g. iproplatin and JM216) have been introduced [6,7], inducing cancer cell death with a mechanism different from that of Pt(II) complexes and remain active against general and cisplatin resistant cancer cell lines [8]. In the present study, two Pt(IV) complexes, containing bidentate nitrogen donor/methyl ligands, with the formula [Pt(X)2Me2(tbu2bpy)] where tbu2bpy = 4,4′-ditert-butyl-2,2′-bipyridine, and X=Cl (**Com1**) or Br (**Com2**) (Fig. 1), serving as the leaving groups have been designed. The interaction between these Pt(IV) complexes and HSA has been investigated, using Ultraviolet-Visible (UV-Vis), fluorescence and CD spectroscopy, as well as, molecular docking simulation.

On the basis of the spectroscopic data, the binding constants, binding sites, and thermodynamic forces between Pt (IV) complexes and HSA were discussed. The obtained results may have important indications in drug delivery and drug design procedures, because HSA is the major protein component of blood plasma and its tertiary structure allows the protein to bind and transport different molecules, including metabolites, gas and exogenous substances such as drugs and diet-derived compounds [9-13].

## MATERIALS AND METHODS

**Materials: **HSA (free fatty acid fraction V, purity > 97%) was obtained from Sigma Chemical Co. and used without further purification. All other reagents were of highest degree of purity and purchased from Merck Chemical Co. The experiments were performed in 5mM Tris buffer pH 7.2, containing 50 mM NaCl (TN buffer). The HSA solutions were used freshly after preparation and the protein concentration was determined from the optical density of appropriate solutions, using the excitation coefficient of 35700 M-1 cm-1 at 280 nm [14].

**General method for the synthesis of Pt complexes: **The Pt(IV) complexes [Pt(X)2Me2(tbu2bpy)], tbu2bpy = 4,4′-ditert-butyl-2,2′-bipyridine and X = Cl, Br, were made by the known methods, using the appropriate platinum(II) complex [PtMe2(tbu2bpy)], and the isolated compounds characterized by comparing their NMR spectra with those of the previously prepared materials [15,16].

**UV-Vis spectroscopy experiments: **The absorption spectra were recorded on a T90+ Spectrophotometer and the quartz cuvette of 1 cm optical path was used. The absorbance titrations were performed in TN buffer (pH 7.2), at 37°C by keeping the concentration of HSA constant (8 µM), while varying the concentrations of each Pt(IV) complex (0-78.4μM). The HSA solutions were freshly prepared just before the measurements. In typical experiments, 1000 µL of HSA solution were placed in the cuvette and the absorbance spectra were recorded between 220 and 350 nm after each addition of the Pt(IV) complexes stock solution. In these experiments, the ligand absorbance was eliminated and the observed absorbance was corrected for dilution. Also, the solutions were allowed to incubate for 5 min, before the absorption spectra were recorded.

**Fluorescence spectroscopy experiments: **Fluorescence spectroscopy studies were carried out on a Cary-Eclipse spectrofluorimeter (Carry-100 Varian, Sydney, Australia). For the ﬂuorescence titration experiments, solution of HSA (1 μM) in TN buffer was titrated with various concentrations of each Pt(IV) complex (0-16.8 μM). The emission spectra were recorded between 300 and 450 nm, with an excitation wavelength of 280 nm. The HSA solutions were freshly prepared just before performing the measurements and the observed fluorescence intensities were corrected for dilution. The band slits for excitation and emission were set at 5 nm. The maximum fluorescence intensity (about 330 nm) was used to calculate the binding constant parameters. To analyze the interaction of HSA and the Pt(IV)complexes, quenching constant (*K*sv) was determined, using the Stern-Volmer equation [17]:

$\frac{F_{0}}{F}=1+K_{q}\tau_{0}[Q]$

(1)

where *F*0 and *F *are relative fluorescence of HSA with and without each Pt(IV) complex, respectively, *K*q is the biomolecular quenching rate constant and *τ*0 stands for the average lifetime of the ﬂuorophore (HSA) in the absence of quencher (*τ*0=10-8 s for most biomolecules). Also, [*Q*] is the concentration of the Pt(IV) complex, and *K*sv refers to the quenching constant. The binding constant and the number of binding sites on HSA were calculated according to the following equation [17]:

$\log\frac{F_{0}-F}{F}=\log K_{b}+\mathrm{nlog}[Q]$

 (2)

Where *F*0 and *F *refer to the fluorescence emission intensities of HSA in the absence and presence of Pt(IV) complexes, respectively, [*Q*] stands for the Pt(IV) complex concentration, *K*b represents the binding constant, and *n *is the number of bound Pt(IV) complex to HSA.

In order to confirm the binding force of the interaction between HSA and Pt(IV) complexes, the thermodynamic parameters were calculated from the van`t Hoff equation:

$\ln K=-\frac{∆H}{\mathrm{RT}}+\frac{∆S}{R}$

(3)

Where *K *is the binding constant at corresponding temperature, *R *represents the gas constant, *T *is the temperature (K) and Δ*H *and Δ*S *are the enthalpy and entropy changes, respectively. Δ*H *and Δ*S *of the reaction were determined from the linear relationship between ln*K *and the reciprocal absolute temperature. The free energy changes of interaction (∆*G*) can be calculated according to the following equation:

∆G=-RTlnK

 (4)

**CD spectroscopy experiments: **The CD spectra after interaction of each Pt(IV) complex with HSA were recorded on an Aviv dichrograph model 215 (Proterion Corp., USA). Far-UV region (195-260 nm) was selected to investigate the HSA secondary structure changes, using 1mm path cuvette. The concentrations of HSA and each Pt(IV) complex were 5 and 21 μM, respectively. All CD spectra were corrected with their corresponding blank solutions, which consisted the same concentration of each Pt(IV) complex in TN buffer, lacking HSA. All measurements were carried out at 293 K with thermostatically controlled cell holder. The instruments were calibrated with ammonium d-10-camphorsulfonic acid. Each spectrum was the accumulation of 5 successive

measurements and the data were expressed as molar residue ellipticity [*θ*] (deg.cm2.dmol-1) which is defined as the following equation [18]:

$\left[\theta \right]=\frac{\mathrm{MRW}\times \theta_{\mathrm{obs}}}{10\times d\times C}$

(5)

Where *MRW *is the mean amino acid residue weight of the HSA (113), *θ*obs is the observed ellipticity in degrees at a given wavelength, *d *is the length of the cuvette in cm and *C *represents the concentration of the protein in mg/ml. The secondary structure alteration of HSA was predicted using CDNN software.

**Molecular modeling: **Molecular docking was carried out using Molegro Virtual Docker software [19]. The structure of each Pt(IV) complex was generated by HyperChem Professional 7.0. Then, energy minimization calculations were carried out at Hartree-Fock (HF) level, using Gaussian 09. The energy minimized Pt(IV) complexes and the simulated HSA were imported to the MVD workspace. The potent binding sites with expanded van der Waals surfaces known as cavities were nominated to extend the grids over the probable binding sites and 5 cavities detected. At a grid resolution of 0.30 Å, the MolDock scoring functions were adjusted as to give 10 final poses. One pose for each ligand suggests the best binding conformation and energy to the cavity 1 with this property; Position: 47.3241 62.4265 60.7493, Volume (Aˆ3): 850.432, Surface (Aˆ2):1876.48. Com1 lays in domain I of HSA. Arg 185, Lys 189, Arg 144, Glu 424, Asp186, Asp 107, Gly 188, Asp 182, Ile 141, Ile 522, Arg 427, Leu 184, Leu 114, Arg 113, Asn 108, Tyr 160, Ser 192 and Glu 519 according to the total energy binding stabilize Com1 in its Binding site by steric bonding interactions. Com2 lays in domain I of HSA. Arg 185, Lys 189, Arg 144, Glu 424, Asp 186, Asp 107, Gly 188, Asp 182, Ile 522, Ile 141, Arg 427, Leu 114, Leu 184, Arg 113, Asn 108, Glu 519, Ser 192 and Tyr 160 according to the total energy binding stabilize Com2 in its Binding site by steric bonding interactions. MDS was performed by GROMACS 4.0.5 package only on chain A of HSA, PDB entry code 2BXD with 3.05 Å resolution [20].

## RESULTS AND DISCUSSION

**Figure 2:**
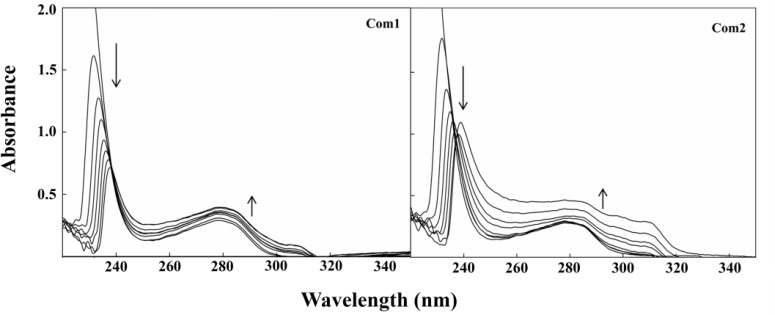
Changes in UV-Vis spectra of HSA with increasing concentrations of Pt (IV) complexes in 5 mM Tris buffer (pH 7.2), containing 50 mM NaCl

**UV-Vis absorption results of the interaction between Pt(IV) complexes and HAS: **Many studies on the interaction of antitumor metal complexes with plasma components, mostly carrier proteins, have been conventionally performed using various spectroscopic techniques, including electronic, vibrational, CD, fluorescence, and NMR spectroscopy. These techniques provide valuable information on the nature and number of protein major sites, participating in the ligand binding, as well as, on the rate, specificity, and reversibility of the interactions [21-25]. UV-Vis spectroscopy which is a useful technique to investigate the structural changes of protein-metal complex formation has been used to analyze the binding of **Com1 **and **Com2 **to HSA. The two main absorption peaks of a typical protein at the wavelengths of 210 nm and 280 nm are generally shaped, respectively by the existence of protein peptide backbone and aromatic amino acids (Trp, Tyr, and Phe). The absorption maximum of HSA at 280 nm depends on the microenvironment in which the aromatic residues are located, albeit this dependence is significantly smaller than in case of fluorescence measurements. As shown in Fig 2, a significant reduction at 210 nm accompanying a red shift was observed upon addition of the increasing concentrations of each Pt(IV) complex to the HSA solution. Also, the intensity of absorption peak at 280 nm demonstrates an enhancement upon the interaction of **Com1 **and **Com2 **with HSA. The results indicated the incidence of protein unfolding with an increase in the hydrophobicity of microenvironment of the aromatic residues, as a result of interaction between HSA and the synthetic Pt(IV) complexes.

**Figure 3:**
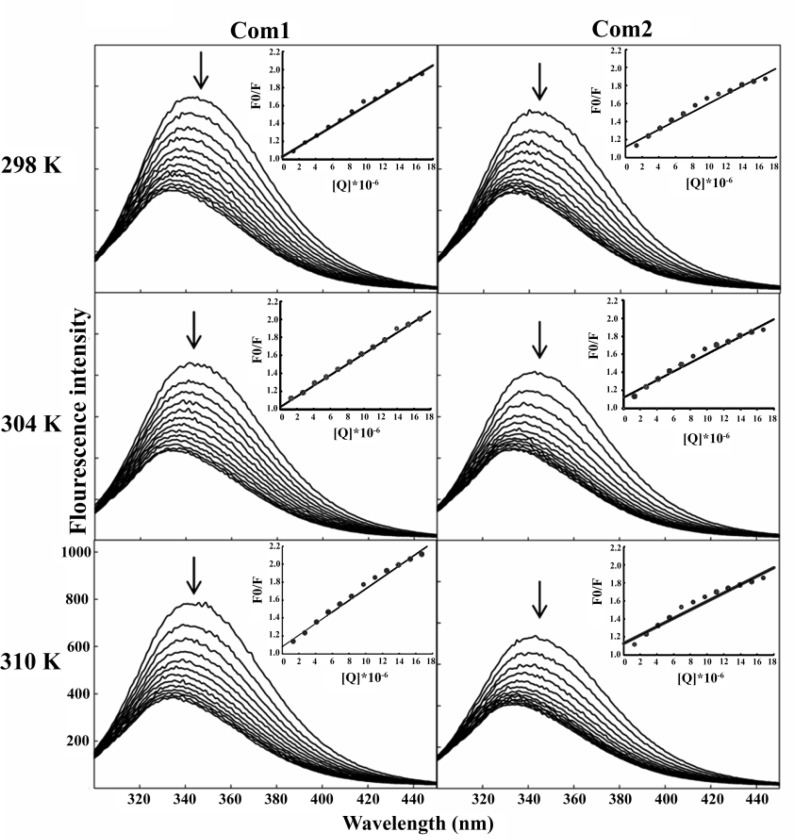
The fluorescence emission spectra of HSA in the presence of various concentrations of the Pt(IV)complexes in 5 mM Tris buffer (pH 7.2), containing 50 mM NaCl at 298, 304 and 310 K. A constant concentration of HSA and different concentrations of Pt (IV) complexes were used. **Inset: **The plot of (F0/F) *vs* [Pt (IV) complexes

**The fluorescence quenching mechanism: **In order to determine the quenching mechanism of the interaction between HSA and the synthetic Pt(IV) complexes, the fluorescence experiments were carried out at three different temperatures (298, 304 and 310 K). Figure 3 demonstrates the gradual reduction of HSA fluorescence intensity with increasing in concentration of each Pt(IV) complex, at 298, 304 and 310 K.

As shown in this figure, the steady blue shift of λmax at all studied temperatures is observed, as Pt(IV) complex concentration increases, indicating that at least part of the Trp residues is transferred/moved into a more hydrophobic environment during the interaction of HSA with Pt(IV) complexes. Also, fluorescence intensity data were analyzed by Eq. 1 to achieve the Stern-Volmer constants (*K*SV), and the plot of *F*0/*F *versus concentration of each Pt(IV) complex. Moreover, the [*Q*] at all temperatures are indicated as the inset of Fig 3 and the Stern-Volmer constant values of the interaction between HSA and Pt(IV) complexes are summarized in Table 1.

**Table 1:** The Stern-Volmer quenching constants for the interaction of HSA and Pt (IV) complexes

**Pt (IV) complex**	**T (K)**	**K** **sv** **×10** **4 ** **(M-1)**	**K** **q** **×10** **12 ** **(M-1S-1)**	**R** **2**
**Com1**	298	7.9	7.9	0.99
	304	8.2	8.2	0.99
	310	9.0	9.0	0.98
**Com2**	298	6.7	6.7	0.97
	304	6.9	6.9	0.97
	310	6.5	6.5	0.96

**Table 2:** The Binding affinity constants and binding number HSA/Pt(IV) complexes (results expressed per protein monomer

**Pt (IV) complex**	**T (K)**	**K** **sv** **×10** **4 ** **(M-1)**	**n**	**R** **2**
**Com1**	298	6.50	0.96	0.99
	304	7.70	0.90	0.99
	310	9.80	0.87	0.99
**Com2**	298	10.15	0.78	0.99
	304	10.13	0.80	0.98
	310	9.30	0.82	0.98

**Table 3:** The thermodynamic parameters for the interaction of Pt (IV) complexes with HSA

**Pt(IV) complex**	**T (K)**	**ΔH** **°** **/kJmol** **–1**	**ΔS** **°** **/Jmol** **-1**	**ΔG** **°** **/kJmol** **–1**
**Com1**	298 304 310	26.85	182.13	- 27.45 - 28.45 - 29.63
**Com2**	298 304 310	- 4.90	79.31	- 28.56 - 29.13 - 29.51

**Table 4:** The HSA secondary structure contents upon interaction with Pt (IV) complexes

	**α-Helix (%)**	**β-Sheet (%)**	**β-Turn (%)**	**Random coil (%)**
**HAS**	66.53	6.17	11.75	15.53
**Com1**	29.74	16.87	15.06	38.32
**Com2**	23.14	20.18	15.92	40.74

The results indicated that the *K*SV values for the binding of **Com1 **to HSA are directly correlated with temperature, suggesting the presence of dynamic collision mechanism. However, in the case of **Com2**, the Stern-Volmer constant did not indicate a clear enhancement, as a result of temperature elevation that is maybe due to restructuration or denaturation effect of **Com2 **on HSA conformation.

**Figure 4:**
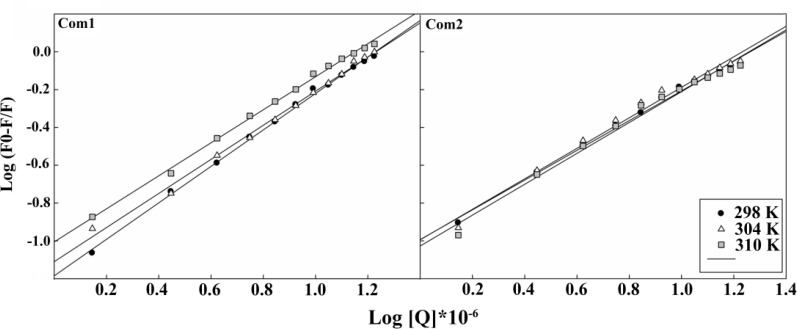
The Plot of log [(F0-F)/F] *vs* log [Pt (IV) complexes] at different temperatures of 298, 304 and 310 K.

**Calculating the binding parameters: **When small molecules bind independently to a set of equivalent site on a macromolecule, the binding constants (*K*b) and the number of binding sites (*n*) could be determined, utilizing Eq. 2. The linear plots of log[(*F*0- *F*)/*F*] versus log[*Q*] for Pt(IV) complexes at 298, 304 and 310 K are shown in Fig 4, and the evaluated *K*b and *n *values are presented in Table 2.

The results indicated the existence of only one class of binding site for each Pt(IV) complex on HSA. These complexes comprise halide leaving groups, displaying different departure rate. The impact of the leaving group departure rates of these synthetic complexes on their anticancer activities and DNA/purine nucleotide binding properties has been already highlighted in our previous publication [26]. However, the results of this study suggested the stronger binding of **Com2 **with HSA in comparison with **Com1**. This result can be explained with the higher hydrophobic character of **Com2 **than **Com1**. The higher hydrophobicity of **Com2 **is to facilitate the easier interaction of this complex with the hydrophobic pocket of HSA in sub-domain IIA. As reported already, the sub-domain IIA also significantly contributes to HSA’s ability to associate with different anionic compounds [27].This also can be explained by shorter distances between Trp and **Com2 **in comparison with **Com1 **(if excitation energy transfer of Trp-Pt(IV) complex contributes to the quenching phenomenon). On the other hand, higher structural flexibility of **Com2 **may facilitate its feasible interaction with HSA. The data in Table 2 suggest that HSA conformation is changed with increasing temperature, therefore, the proteins binding properties can change as a function of temperature elevation.

**Thermodynamic parameters and the nature of binding forces: **The interaction forces involved in binding of small ligands with biomacromolecules are usually classified into hydrogen-bond formation, van der Waals, electrostatic and hydrophobic forces [28]. By plotting the binding constants, *K*b, in Table 2, versus temperature (data are not shown) and using Eqs 3 and 4, the thermodynamic parameters were obtained (Table 3**)**.

The negative values of ΔG indicated that the binding interactions between the Pt(IV) complexes and HSA were spontaneous. On the other hand, the positive values of ΔH and ΔS suggested that hydrophobic interactions and electrostatic attractions were the major contributing forces in binding of **Com1 **to HSA. The positive value of ΔS and negative value of ΔH demonstrated the significant role of hydrophobic/hydrogen bonding forces in **Com2**/HSA interactions [29].

**Figure 5:**
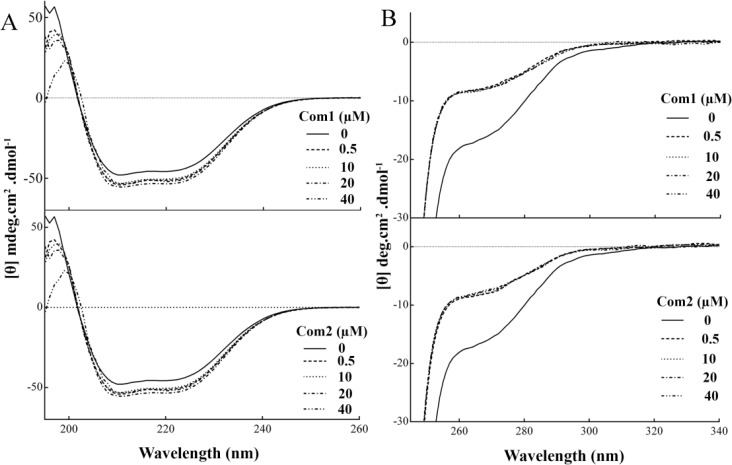
Circular dichroism analysis of HSA after interaction with the synthetic Pt complexes.

**CD spectroscopy results: **To investigate the possible structural alteration of HSA due to interaction with Pt(IV) complexes, both far and near-UV CD analysis were utilized. The far-UV CD spectra of HSA in the presence and absence of these Pt(IV) complexes are showed in Fig 5A. As shown in this figure, the CD spectrum of HSA exhibited two negative bands at 208 and 222 nm, which are characteristic of the protein α-helix and β-sheet contents, respectively. After addition of each Pt(IV) complex, the intensity at 208 and 222 nm were significantly more negative, indicating the significant changes in the protein secondary structure. The far-UV CD spectra of HSA were deconvulated and the obtained results are indicated in Table 4.

As shown, in the free state of HSA, the secondary structure consisted of 66.53% α- helix, 6.17% β-sheet, 11.75% β-turn and 15.53% random coils. However, after addition of these Pt(IV) complexes, the secondary structure of the protein was widely altered. With increasing the complex concentration, the amount of α-helix was reduced, and at the same time the content of other structures increased. The results indicated that these Pt(IV) complexes were capable to induce significant structural changes in HSA. Also the results of near UV-CD (Fig. 5B), notify that both complex interact with HSA and induce protein's structural alteration in similar level. As shown in Fig 5B, the wavelength region of 280-295 nm (a region that protein backbone have no contribution), there is a significant induction in CD signals, corresponding to the interaction of the **Com1 **and **Com2 **with the binding site of protein. The majority of the

CD changes are due to the induced chirality in the bound **Com1 **and **Com2 **molecules.

A & B stand for the Far & Near-UV CD spectra of HSA in presence these complexes, respectively.

**Figure 6:**
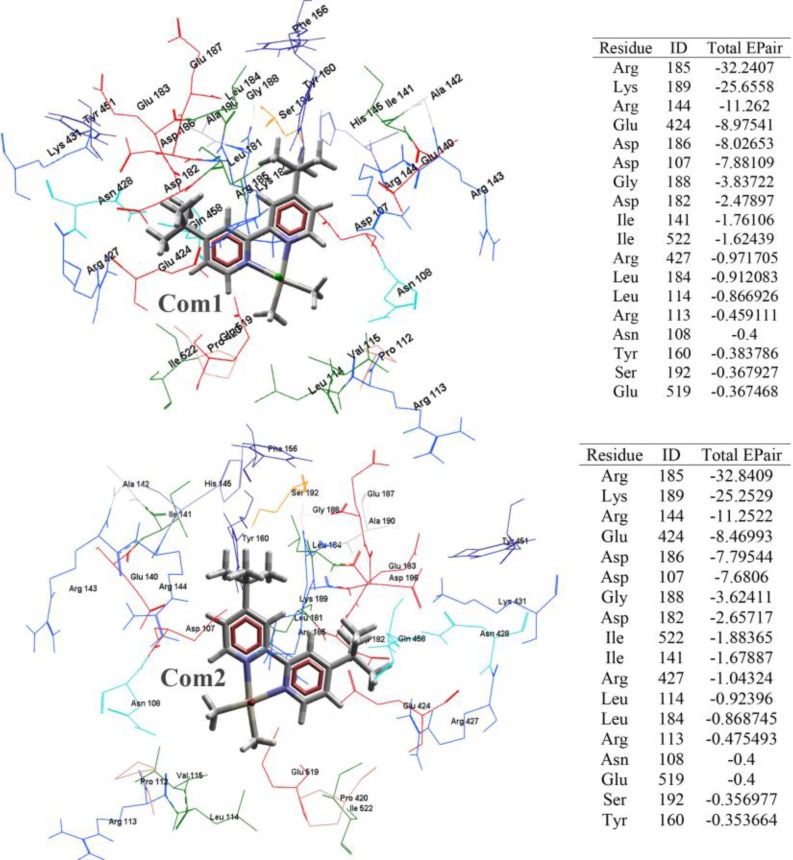
Molecular docking simulation of the Pt (IV) complexes with HSA

**Molecular docking results: **The blind docking mode with the lowest binding free energy is shown in Fig 6. **Com1 **and **Com2 **are surrounded by 18 residues which are shown in Figure 6.

The interaction occurs in the area between sub-domains IB and domain III, but mainly in IB. The data indicated that Arg-185, Lys-189 in sub-domain IB interact effectively with **Com1**. The same residues as for **Com1 **have been detected to interact with **Com2**. ∆G binding for **Com1 **and **Com2 **with HSA respectively are -108.508 (kJ/mol), -108.183 (kJ/mol). The negative values of ΔG for the interaction of **Com1 **and **Com2 **with HSA indicated that the binding interactions between the Pt(IV) complexes and HSA were spontaneous that is in consistent with the results of fluorescence measurements. The ΔG values for both complexes are literally the same, demonstrated that both Pt(IV) complexes interact with HSA in the same way.

In conclusion, this study suggests that variation in the leaving group, displaying differing departure rate, has no important contribution in denaturing effects of the Pt(IV) complexes against HSA. The obtained results might be noteworthy on basis of understanding the relationship between structure and susceptibility for development of severe side effects which are associated with the clinical application of currently in use platinum-based drugs.

## Notes

**Acknowledgments: **This work was supported by Iran National Science Foundation (grant nos. 92001695 and 90005876). Also, the supports of research council of Shiraz University are acknowledged.
